# Evaluation of a Mobile Telesimulation Unit to Train Rural and Remote Practitioners on High-Acuity Low-Occurrence Procedures: Pilot Randomized Controlled Trial

**DOI:** 10.2196/14587

**Published:** 2019-08-06

**Authors:** Jennifer Jewer, Michael H Parsons, Cody Dunne, Andrew Smith, Adam Dubrowski

**Affiliations:** 1 Faculty of Business Memorial University St John's, NL Canada; 2 Faculty of Medicine Memorial University St John's, NL Canada; 3 Faculty of Health Sciences Ontario Tech University Oshawa, ON Canada

**Keywords:** medical education, distributed medical education, simulation training, emergency medicine, rural health, remote-facilitation, assessment, chest tubes

## Abstract

**Background:**

The provision of acute medical care in rural and remote areas presents unique challenges for practitioners. Therefore, a tailored approach to training providers would prove beneficial. Although simulation-based medical education (SBME) has been shown to be effective, access to such training can be difficult and costly in rural and remote areas.

**Objective:**

The aim of this study was to evaluate the educational efficacy of simulation-based training of an acute care procedure delivered remotely, using a portable, self-contained unit outfitted with off-the-shelf and low-cost telecommunications equipment (mobile telesimulation unit, MTU), versus the traditional face-to-face approach. A conceptual framework based on a combination of Kirkpatrick’s Learning Evaluation Model and Miller’s Clinical Assessment Framework was used.

**Methods:**

A written procedural skills test was used to assess Miller’s learning level— *knows* —at 3 points in time: preinstruction, immediately postinstruction, and 1 week later. To assess procedural performance (*shows how*), participants were video recorded performing chest tube insertion before and after hands-on supervised training. A modified Objective Structured Assessment of Technical Skills (OSATS) checklist and a Global Rating Scale (GRS) of operative performance were used by a blinded rater to assess participants’ performance. Kirkpatrick’s *reaction* was measured through subject completion of a survey on satisfaction with the learning experiences and an evaluation of training.

**Results:**

A total of 69 medical students participated in the study. Students were randomly assigned to 1 of the following 3 groups: comparison (25/69, 36%), intervention (23/69, 33%), or control (21/69, 31%). For *knows*, as expected, no significant differences were found between the groups on written knowledge (posttest, *P*=.13). For *shows how*, no significant differences were found between the comparison and intervention groups on the procedural skills learning outcomes immediately after the training (OSATS checklist and GRS, *P*=1.00). However, significant differences were found for the control versus comparison groups (OSATS checklist, *P*<.001; GRS, *P*=.02) and the control versus intervention groups (OSATS checklist, *P*<.001; GRS, *P*=.01) on the pre- and postprocedural performance. For *reaction*, there were no statistically significant differences between the intervention and comparison groups on the satisfaction with learning items (*P*=.65 and *P*=.79) or the evaluation of the training (*P*=.79, *P*=.45, and *P*=.31).

**Conclusions:**

Our results demonstrate that simulation-based training delivered remotely, applying our MTU concept, can be an effective way to teach procedural skills. Participants trained remotely in the MTU had comparable learning outcomes (*shows how*) to those trained face-to-face. Both groups received statistically significant higher procedural performance scores than those in the control group. Participants in both instruction groups were equally satisfied with their learning and training (*reaction*). We believe that mobile telesimulation could be an effective way of providing expert mentorship and overcoming a number of barriers to delivering SBME in rural and remote locations.

## Introduction

### Challenges Accessing Simulation-Based Medical Education

The provision of acute care in rural and remote areas presents unique challenges. Skills related to high-acuity low-occurrence procedures and clinical encounters are particularly susceptible to degradation over time and are inadequately served through on-the-job experience alone [1]. Therefore, a systematic approach to training personnel for these procedures is required. In recent years, an increasing proportion of this training has made use of simulation-based modalities. Simulation-based medical education (SBME) has been shown to be an effective training approach because it can provide opportunities to practice infrequently encountered procedures [2-5] without compromising patient safety [6]. However, SBME often takes place in urban centers, and it can be difficult for rural and remote acute care practitioners to access these centers because of geographic, cost, and time constraints [7,8].

SBME delivered through technologies such as telesimulation and mobile simulation has been shown to be an effective means of training medical practitioners and has helped to address some of the above constraints [4,7-17]. However, use of these technologies is accompanied by their own challenges. Telesimulation involves delivering SBME over the internet, but effective delivery of telesimulation training can be limited if the trainees are unable to access simulation equipment or an efficient training setup. Mobile simulation can address constraints by delivering an immersive simulation environment in a purposefully designed unit. However, mobile simulation often involves bringing an expert to rural and remote sites to facilitate the session. This can often prove to be quite expensive and prohibitive because of time constraints.

Through an iterative design process, our multidisciplinary group has developed an MTU that explores many of the challenges to the delivery of SBME to rural and remote acute care practitioners. The intention is the deployment of the MTU at a rural or remote location that could house the skills training session through communication with an off-site, skilled mentor. Such a deployment would provide trainees with the appropriate simulation equipment, a standardized training environment, and access to an experienced mentor to guide the training. To our knowledge, this is one of the few such units, which combines telecommunication and mobile simulation to deliver such training.

A rigorous, theory-based, iterative approach was followed to develop the MTU and evaluate the acceptability and feasibility of delivering training remotely using the unit. Details on the development of the MTU and training materials have been published elsewhere [18-23].

The objective of this study was to compare the educational efficacy of face-to-face versus remote delivery of educational content with respect to learner’s perceptions and objective assessment of procedural performance.

### Framework for Learning Assessment

**Figure 1:**
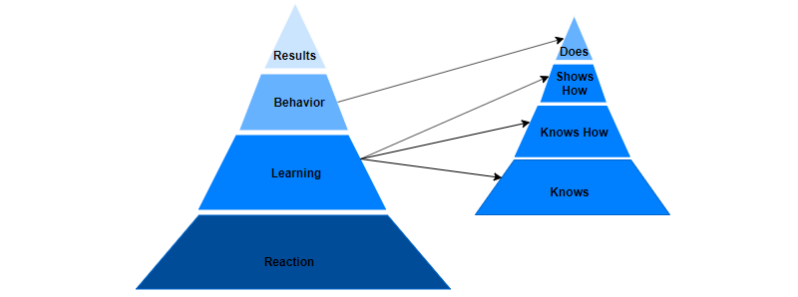
Framework for Learning Assessment, based on Kirkpatrick (left) and Miller (right). Adapted from Dubrowski et al [26].

This study uses a conceptual framework based on a combination of Kirkpatrick’s Learning Evaluation Model [24] and Miller’s Clinical Assessment Framework [25] to guide the assessment of the MTU. This model (Figure 1; adapted from Dubrowski et al [26]) is based on the work of Moore et al [27] who developed a framework “of an ideal approach to planning and assessing continuing medical education that is focused on achieving desired outcomes” (pg 3). The new model incorporates Kirkpatrick’s 4 levels, which represent a sequence of ways to evaluate a program, with Miller’s assessment tools for each level of competence.

The base of Kirkpatrick’s model relates to subject *reaction*, measuring how participants react to or perceive program content. There is no direct correlation of this feature to a level on Miller’s framework. The second level of the Kirkpatrick model, *learning*, corresponds to the bottom 3 levels of Miller’s framework (*knows*, *knows how*, and *shows how*), whereas the third level of Kirkpatrick’s framework, *behavior*, is closely related to the top of Miller’s framework, *does*. Finally, the top level of Kirkpatrick’s model, *results*, does not relate to Miller’s framework. This study examines Kirkpatrick’s *reaction* and *learning*, consisting of *knows* and *shows how*. We do not examine *knows how* because of anticipated challenges of subject retention and expected loss to follow-up during the study. Rather, we decided to measure the higher level *shows how* because we could evaluate the participants’ performance of the procedure during the study. We do not examine Kirkpatrick’s *behavior* and, consequently, do not examine Miller’s *does*. We also do not examine Miller’s *results*, as these are assessments of practice in a clinical setting, and this study is limited to an experimental setting. This paper discusses the findings in relation to Kirkpatrick’s *reaction* and *learning* (consisting of Miller’s *knows* and *shows how*) levels.

## Methods

### Research Setting

This study was conducted at Memorial University of Newfoundland. Training of rural and remote acute care practitioners is of particular interest in the province, as 40% of the population lives in rural areas, and the province has a relatively small population (525,000) distributed across a large geographic area (405,000 km^2^). Acute care is delivered at a variety of health centers and hospitals across the province. These sites are staffed by physicians, nurses, and nurse practitioners with varying levels of experience. Access to SBME opportunities is often limited. Health Research Ethics Board of Memorial University of Newfoundland approved this study.

**Figure 2:**
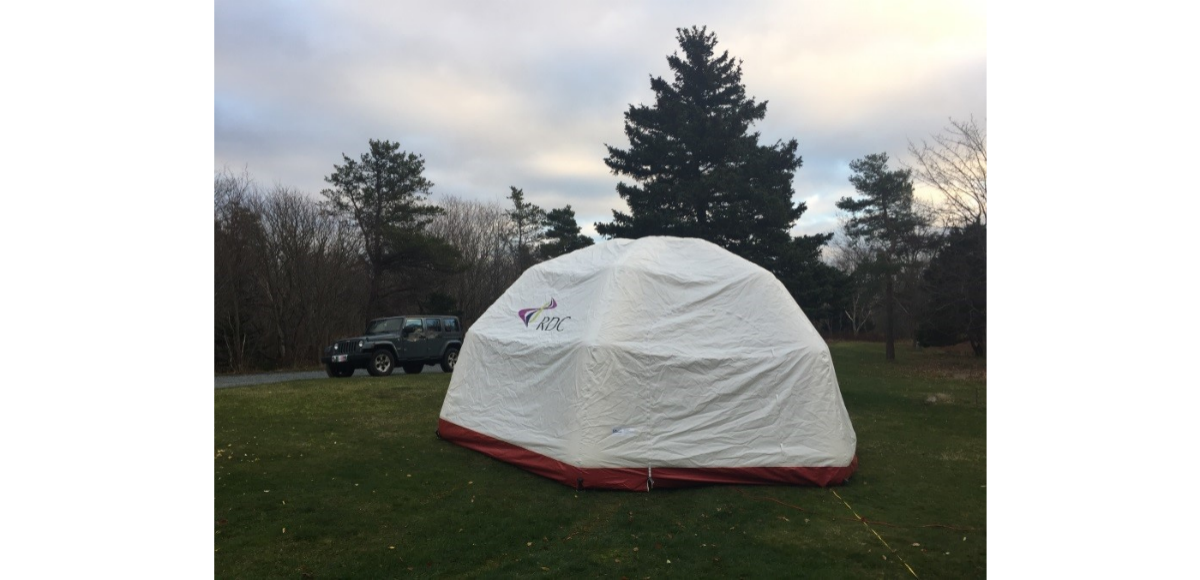
The mobile telesimulation unit rapid deployment tent.

**Figure 3:**
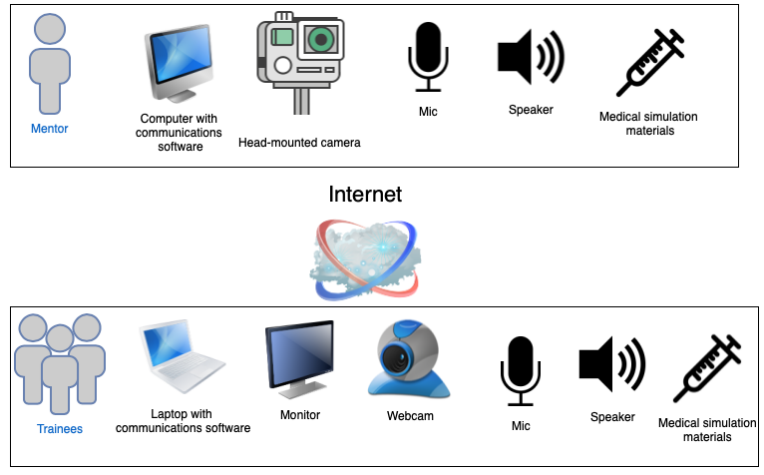
Overview of the setup for the mentor and the trainees in the mobile telesimulation unit.

**Figure 4:**
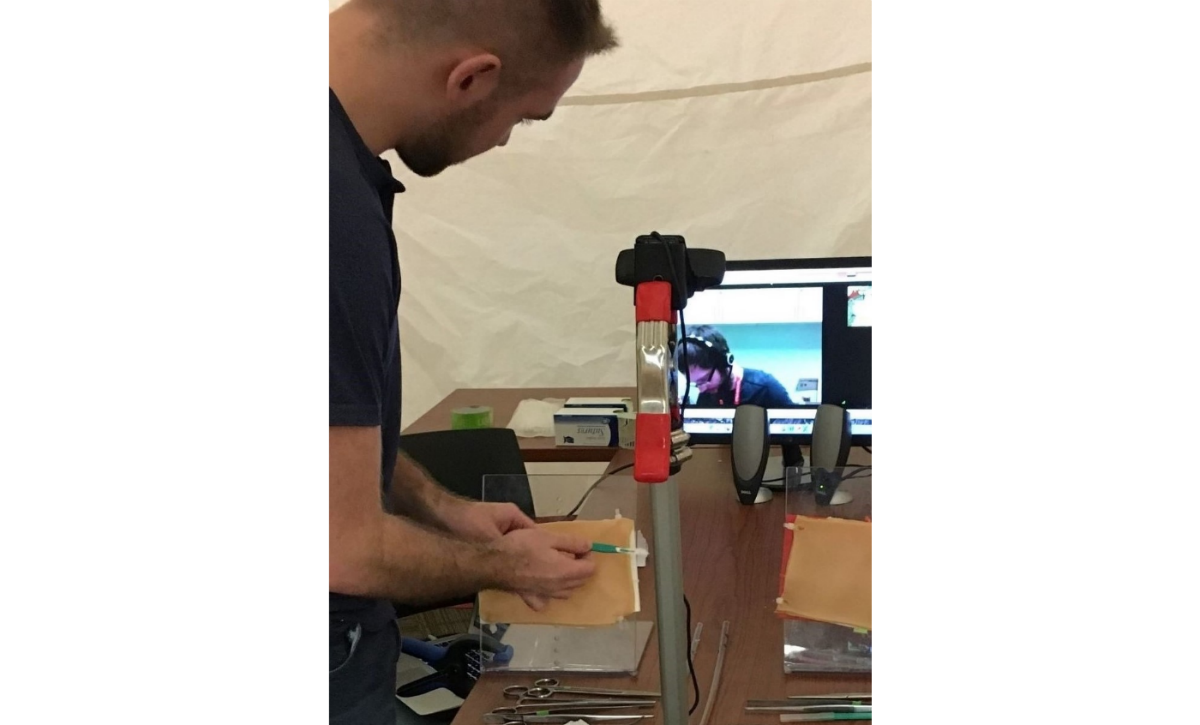
The interior of the mobile telesimulation unit demonstrating setup for procedural training.

The MTU consists of an inflatable rapid deployment tent (Figure 2), which is outfitted with portable technology necessary to allow for 2-way communication between the trainees and the mentor: laptop with communications software, monitor, camera, speaker, and microphone and a portable wireless internet hub. The mentor uses comparable software, a camera, speaker, and microphone to communicate with the trainees. Off-the-shelf and low-cost equipment was used to keep the design of the MTU accessible and practical. Both the trainees and the mentor would have similar simulation supplies and setup to enable efficient demonstration and instruction (Figures 3 and 4). Studies by Jewer et al provide more information on the MTU [18,21].

The eventual goal was to deliver simulation-based training remotely through the use of a self-contained vehicle outfitted with simulation equipment necessary for delivery of a number of scenarios. However, for the purpose of our test-of-concept approach, a portable and rapid deployment tent was used.

### Study Design

A randomized controlled trial design was followed. A total of 3 sessions were held to compare the learning outcomes of participants who received training remotely in the MTU versus those who received the same training face-to-face. To minimize variables affecting study outcomes, face-to-face training sessions also took place in the MTU space. A control group (ie, received no training) was included to show that the intervention group (ie, remote) was not inferior to the comparison group (ie, face-to-face), and that both instructional approaches are actually effective [28].

The sessions focused on teaching an important high-acuity low-occurrence procedure, chest tube insertion, using a low-fidelity setup: 3D-printed ribs, secured to a plexiglass stand, covered with low-cost simulated skin, and subcutaneous tissue (Figure 4). The chest tube insertion was selected as a representative procedure because it is an essential skill in acute care settings requiring precision [29], and it is a multistep procedure amenable to objective scoring. The training sessions were 20-min long and consisted of simulation-based training, with deliberate hands-on practice and mentor feedback.

**Figure 5:**
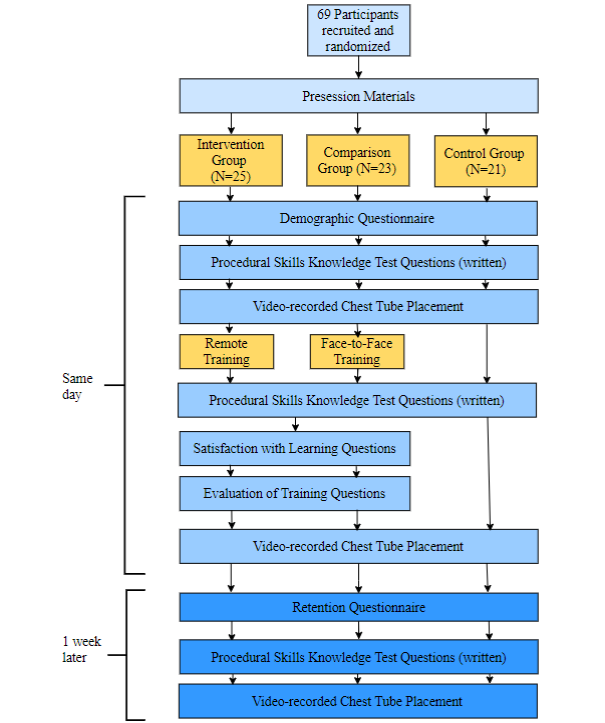
Study design.

Figure 5 depicts the flow of the study procedure. A week before the procedural session, participants were emailed presession information consisting of a Web-based New England Journal of Medicine video, demonstrating proper performance of the procedure and important details about chest tube insertion including indications, contraindications, complications, and necessary equipment [30]. This was to help ensure that participants started with a similar base level of knowledge.

Participants were randomly assigned to 1 of 3 groups: intervention, comparison, and control. Testing procedures were conducted before the training (pretest), after the training (posttest), and 1 week later (retention test). During the pretest, participants completed a questionnaire on demographic information, the number of times they performed or witnessed a chest tube insertion before this session, their previous experience with SBME, and their previous experience with telemedicine. Next, participants completed a written procedural skills knowledge test on a number of chest tube procedure-specific questions. The demographic questionnaire and the procedural skills knowledge test were written components used to assess whether there were differences in the baseline knowledge about the chest tube procedure within or between the groups at the start of the study. The procedural skills knowledge test was also used to measure learning after the session. This corresponds to the *knows* level of learning. These materials were reviewed by an experienced emergency medicine physician to determine if differences existed.

**Figure 6:**
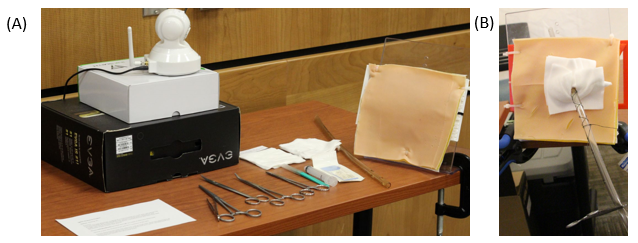
Setup used in the video recording of the chest tube procedure (A) and example of a completed chest tube insertion (B).

To measure *shows how*, during the pretest, participants were video recorded performing a chest tube insertion on a low-fidelity simulated model (Figure 6). A modified Objective Structured Assessment of Technical Skills (OSATS) checklist and a Global Rating Scale (GRS) of operative performance were used to assess procedural performance [31].

After the training session, during the posttest, participants in the intervention and comparison groups were asked to evaluate their satisfaction with learning and their evaluation of the training. This corresponds to Kirkpatrick’s *reaction* level of the learning framework. Participants also completed the written procedural skills knowledge test again (ie, *knows*). All participants were then once again video recorded performing a chest tube insertion (ie, *shows how*).

Furthermore, 1 week after the training session (retention test), the participants completed a questionnaire on their experiences with the procedure in the past week. They also completed the written procedural skills knowledge test again (ie, *knows*), and they were video recorded for the third time performing a chest tube insertion (ie, *shows how*).

An emergency medicine physician with 11 years of clinical emergency room experience used the modified OSATS checklist and GRS to assess the participants’ performance on the video recordings. The reviewing physician was blinded to participants’ identity and was unaware of the phase of the study (pretest, posttest, or retention test). Overall, 12% of the videos were randomly selected for review by a second experienced emergency medicine physician. The modified OSATS checklist and GRS scores were used as the primary indicators of learning outcomes (ie, *shows how*).

### Participants

Medical students during their first and second year of training (approximately 80 students per cohort) were invited to participate in the study. Participation was voluntary and was limited by the number of slots available at a scheduled data collection time (Multimedia Appendix 1). These medical students were novices in the chest tube procedure, and using such subjects with similar background knowledge and skills enabled us to more clearly measure learning. Participants provided informed consent before enrollment in the study.

### Measures

#### Learning—Knows

To measure the *knows* dimension of learning, participants were asked a set of chest tube procedure–specific questions (Textbox 1).

**Procedural skills knowledge test questions (possible score: 15).**

- Name 3 indications for chest tube placement.
- Name 3 contraindications to chest tube placement.
- Name 4 potential complications of chest tube placement.
- Name 5 essential pieces of equipment for chest tube placement.

#### Learning—Knows How

Participants’ performance of the chest tube procedure was evaluated using a modified OSATS checklist to measure the *knows how* dimension of learning. The OSATS checklist was originally developed and validated to assess the performance of multiple surgical procedures at different stations [31]. It has since been used to assess the performance of a single surgical procedure [32]. Research has demonstrated that the OSATS has high reliability and construct validity for measuring technical abilities outside of the operating room [31].

**Figure 7:**
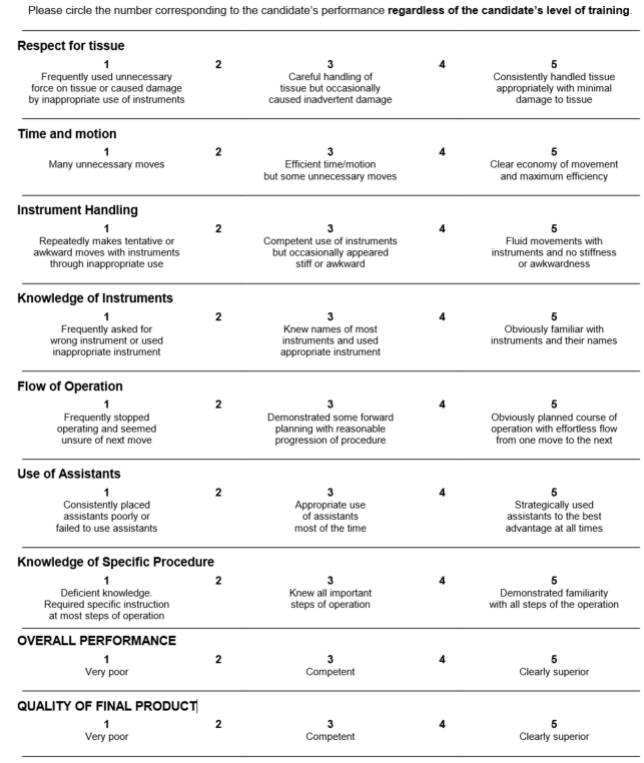
Global Rating Scale of operative performance.

This study used a modified OSATS checklist and a GRS of operative performance. The checklist consists of 10 items that are scored as done correctly or not (Textbox 2). For the purposes of this study, 1 item of the scale was removed because it was not relevant to our training scenario (ie, item #9—a Pleur-evac setup was not available to participants). The GRS is composed of 9 items, each measuring a different aspect of operative performance. Each item was graded on a 5-point Likert scale from 1, poor performance, to 5, good performance (Figure 7). Again, for the purposes of this study, 2 items of the scale were removed because they were not appropriate for the training scenario (removed items included *use of assistants* and *knowledge of instruments* because there was no assistant in the study design and knowledge of instruments implied participants were *asking* for the right things or *saying* the right names, something which was not part of the study design). Thus, the maximum GRS score attainable is 35 points, and the minimum is 7 points.

**Checklist for chest tube insertion (not done, incorrect=0; done, correct=1).**

- Injects local anesthetic
- Cuts skin with scalpel to subcutaneous tissue plane (no scything)
- Uses blunt dissection to enter chest cavity
- Enters pleural space above rib
- Checks position with digit before inserting chest tube
- Inserts chest tube safely using Kelly at the tip of the tube
- Inserts correct length of chest tube into chest
- Secures chest tube to chest wall with silk or nylon
- Connects tube and secures to drainage system with tape
- Applies airtight dressing

#### Reaction

To measure participants’ reactions to the training, participants in the remote and comparison groups were asked to evaluate the training by indicating whether they thought the MTU could play an important role in rural and remote medical training, how satisfied they were with their overall experience in the MTU, and if they would recommend the MTU approach to their colleagues. Participants were also asked to indicate their satisfaction with the learning experiences. These measures were adapted from the National League of Nursing (NLN) Student Satisfaction and Self-Confidence in Learning scales [33]. These NLN scales have been widely used and have been found to have sufficient reliability and validity to be used in education research [33,34].

### Data Analysis

Participants were assigned a unique identifier, and this was used to anonymize the data before analysis with respect to their training group. Data analysis was completed using SPSS version 25. Descriptive statistics were computed for the demographic variables.

#### Learning—Knows

Because our data did not enable us to use the parametric repeated measures analysis of variance to analyze the pretest, posttest, and retention written procedural skills tests, we created 2 new variables (pretest minus posttest score and posttest minus retention test score). The Kruskal-Wallis test (nonparametric equivalent) was then used to compare participants’ performance on the procedural skill test between the groups.

#### Learning—Knows How

There was acceptable interrater reliability between the 2 raters who evaluated the performance of the chest tube procedure. An excellent intraclass correlation coefficient (ICC) of 0.909 was found for the GRS, and a good ICC of 0.757 was found for the checklist. Again, limited to nonparametric techniques, we created 2 new variables: 1 variable to calculate the difference between the pre- and postchecklist and GRS scores, and the second to calculate the difference between the postchecklist and retention checklist and GRS scores. A Kruskal-Wallis test was then used to compare pretest, posttest, and retention test scores for the 3 groups (ie, intervention, comparison, and control) on the modified OSATS checklist and GRS scores.

#### Reaction

The Mann-Whitney *U* test (nonparametric equivalent) was used to compare the intervention with the comparison groups on satisfaction with learning and their evaluation of the training.

For all tests, a *P* value less than .05 was considered statistically significant.

## Results

In total, 69 medical students participated in the study across the 3 different sessions (Table 1). Participants were randomly assigned to their study group: intervention, comparison, or control groups.

### Participants’ Experience

The groups were very similar—mean age in low to mid-20s and relatively equally mixed between the first and second year of medical school. If there was any impact on results of students being in the first or second year of medical school, it would probably negatively influence the intervention group because a slightly higher percentage of participants in this group were in their first year. However, training on chest tube insertion is not part of the standard curriculum in the first 2 years of medical school, and most participants indicated that they had never performed or even witnessed a chest tube placement before; therefore, the presession materials and this training were the first exposures to the skill for most participants. The majority had participated in low-fidelity SBME using task trainers before, between 1 and 10 times, and the majority had never received training using telemedicine.

**Table 1:** Participants’ experience.

Characteristics		Intervention group (n=25)	Comparison group (n=23)	Control group (n=21)
Age (years), mean		25	23	21
**Level of medical training, n (%)**				
	1st year	16 (64)	6 (26)	9 (43)
	2nd year	9 (36)	17 (74)	12 (57)
**Performed a chest tube insertion before, n (%)**				
	Never	24 (96)	22 (96)	20 (95)
	Yes	1 (4)	1 (4)	2 (5)
**Witnessed a chest tube insertion before, n (%)**				
	Never	22 (88)	20 (87)	15 (71)
	Yes	3 (12)	3 (13)	6 (29)
**Participated in simulation-based medical education^a^, n (%)**				
	Never	2 (8)	5 (22)	4 (19)
	1-10 times	21 (84)	18 (78)	15 (71)
	>10 times	2 (8)	0 (0)	2 (9.5)
**Past exposure to telemedicine, n (%)**				
	Never	25 (100)	18 (78)	19 (91)
	At least quarterly	0 (0)	5 (22)	2 (10)

^a^Low-fidelity task trainers (eg, suturing pads, airway models, and chest tube placement).

**Table 2:** Questionnaire responses at the time of retention test (1 person from the comparison group and 2 from the control group did not complete the retention test).

Characteristics	Intervention group (n=25)		Comparison group (n=22)		Control group (n=19)	
	No, n (%)	Yes, n (%)	No, n (%)	Yes, n (%)	No, n (%)	Yes, n (%)
Performed a chest tube in the past week	23 (92)	2 (8)	22 (100)	0 (0)	19 (100)	0 (0)
Witnessed a chest tube in the past week	25 (100)	0 (0)	22 (100)	0 (0)	19 (100)	0 (0)
Received any training or done further reading on chest tube insertions in the past week	24 (96)	1 (4)	21 (96)	1 (4)	17 (90)	2 (11)

Similarly, the retention test survey, assessing exposure to chest tube insertions in the week since the training, showed no real differences between the groups. Most had not performed a chest tube since the training, witnessed a chest tube, or received any training or done any further reading on chest tube insertions (Table 2).

### Learning—Knows

A Kruskal-Wallis test was used to compare the results of the procedural skills knowledge test. This was a brief written test completed after receiving the presession materials but before the training session. The mean test score (out of a possible score of 15) and SD were 11.52 (2.07) for the intervention group, 10.91 (2.02) for the comparison group, and 10.76 (2.56) for the control group. There was no significant difference between the groups before starting the session (χ^2^_2_=1.9; *P*=.39). This indicates that the participants in the 3 groups had similar levels of written knowledge about chest tube insertion before the training.

Subsequent Kruskal-Wallis tests revealed that there were no significant differences between groups from the pretest to the posttest (χ^2^_2_=4.1; *P*=.13) or from the posttest to the retention test (χ^2^_2_=1.6; *P*=.46; Multimedia Appendix 2).

### Learning—Knows How

**Figure 8:**
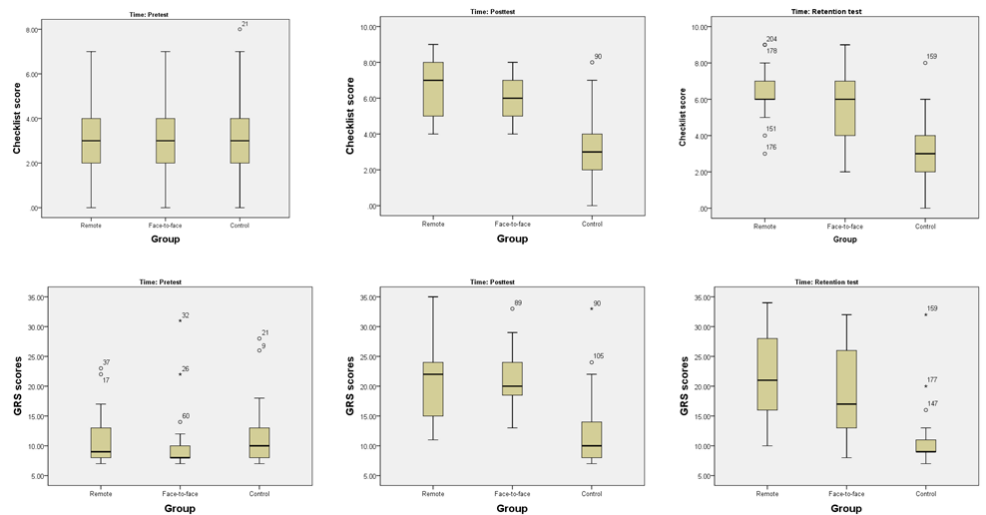
Box plots of the modified Objective Structured Assessment of Technical Skills checklist and GRS scores. GRS: Global Rating Scale.

A total of 204 videos of procedural performance were included in the analysis, with 3 videos per participant (3 participants did not complete the retention test). Results of the modified OSATS checklist and GRS assessment for the 3 groups (pretraining, posttraining, and 1 week after the training) are shown in Multimedia Appendix 3. Box plots of the scores are shown in Figure 8.

A Kruskal-Wallis test revealed that there were statistically significant differences between the groups on the pre- and post-OSATS checklist and GRS scores (Multimedia Appendix 4). Pairwise comparisons were performed using Dunn [35] procedure with a Bonferroni correction for multiple comparisons. This post hoc analysis revealed statistically significant differences in median OSATS checklist and GRS scores differences between the control and comparison and the control and intervention groups, but not between the comparison and intervention groups. There was no difference between the posttest and retention scores.

### Reaction

Satisfaction with learning and evaluation of the training measures was used to examine participants’ reaction to the training.

#### Satisfaction with Learning

The results of the satisfaction with learning questions (adapted from the NLN scales) that were asked in the posttest for the intervention and comparison groups are shown in Table 3. On average, participants rated the teaching methods as helpful and effective for the intervention and comparison groups, with scores 4.52 and 4.65, respectively, out of 5. Averaged responses also indicated that they enjoyed how the teacher taught the session for the intervention and comparison groups with scores 4.40 and 4.52, respectively, out of 5. A Mann-Whitney *U* test revealed that there were no statistically significant differences between the intervention and comparison groups on these items.

**Table 3:** Self-reported learning—scale of 1 (strongly disagree) to 5 (strongly agree).

Measurement item (satisfaction with learning)	Intervention group (n=25), mean (SD)	Comparison group (n=23), mean (SD)	Mann-Whitney *U* test		
			*U*	z	*P* value
The teaching methods used were helpful and effective.	4.52 (0.71)	4.65 (0.49)	306.5	0.47	.65
I enjoyed how the teacher taught the session.	4.40 (0.82)	4.52 (0.59)	299.0	0.27	.79

#### Participant Evaluation of Training

Participants in the intervention and comparison groups were asked to evaluate their experiences with the training session that took place physically in the MTU space. Participants indicated that the MTU could play an important role in rural medical training (4.32 and 4.48 out of 5 for the intervention and comparison groups, respectively), they were satisfied with their overall experience in the MTU (4.32 and 4.43 out of 5 for the intervention and comparison groups, respectively), and they would recommend the MTU to their colleagues for SBME (4.32 and 4.43 out of 5 for the intervention and comparison groups, respectively). A Mann-Whitney *U* test revealed that there were no statistically significant differences between the intervention and the comparison groups on any of these questions (Table 4).

**Table 4:** Participants’ evaluation of training modality—scale 1 (strongly disagree) to 5 (strongly agree).

Evaluation of training modality	Intervention group (n=25), mean (SD)	Comparison group (n=23), mean (SD)	Mann-Whitney *U* test or *t* test		
			*U*	z	*P* value
Do you think the MTU^a^ could play an important role in rural medical training?	4.32 (1.11)	4.48 (0.51)	276.0	−0.27	.79
How satisfied are you with your overall experience in the MTU?	4.32 (0.56)	4.43 (0.59)	319.5	0.38	.45
Would you recommend the MTU to your colleagues for simulation-based medical training?	4.32 (0.56)	4.43 (0.73)	331.0	1.02	.31

^a^MTU: mobile telesimulation unit.

## Discussion

### Principal Findings

Using a conceptual framework based on Kirkpatrick’s and Miller’s works [24,25], we examined learning based on *knows* and *shows how* levels and also studied the *reactions* of the participants to the training. We found this framework useful to help ensure a thorough evaluation of the training delivered using an MTU. The results from this study indicate comparable learning (*knows* and *shows how*) and *reactions* of participants who received the procedural skills training remotely with those who received the training face-to-face.

Consistent with the literature, we found that subject’s knowledge level (*knows*) remained unchanged after the training. This is what was expected as there are 2 distinct key areas of knowledge with respect to competent procedural skills performance—one related to factual background information (*knows*) and the second being the ability to complete all necessary steps (*shows how*). Our study focused on *shows how*, as the ability to physically and capably complete a procedural skill relies on deliberate practice of that skill [36]. Nevertheless, it was important to measure the procedural skill knowledge (*knows*), as it enabled us to ensure there was a consistent knowledge level across all groups. This is particularly important, as procedural skills training sessions aim to enable participants’ performance of the procedure (ie, *shows how*), which is a higher level than *knows*.

With respect to the *shows how* learning, our study supports previous findings related to telesimulation and mobile simulation [7,8,37,38]. We found that the learning outcomes for the participants who received training remotely through the MTU, as assessed using modified OSATS checklist and GRS, are comparable with those of the face-to-face simulation-based training group. Furthermore, participants who received training, either remotely or face-to-face, received statistically significantly better scores than those who did not receive instruction (ie, the control group). The average scores on the checklist more than doubled from the pretests to posttests for the intervention (from 3 to 6.54) and comparison groups (from 2.96 to 6.22). However, the increase in the scores for the control group was negligible, increasing by only 0.33 points (from 2.91 to 3.24). This indicates that training resulted in similar acquisition of skills-based knowledge for both the remote training and face-to-face groups.

Retention tests indicated that there were no statistically significant differences in skills retention between all 3 of the groups. On average, differences between the scores on retention test–modified and posttest-modified OSATS checklist and GRS scores either stayed the same or decreased slightly for all groups. From this, we conclude that the manner of instructional delivery (either remote or face-to-face) does not impact retention.

In addition to the comparable learning outcomes, participants had similarly high levels of satisfaction with learning in the MTU. Rating the teaching methods as helpful and effective, participants indicated that, on average, they enjoyed instruction during the session. This is encouraging as satisfaction with the training, in the case of the MTU concept facilitated through a local healthcare facility, could influence commitment and readiness to transfer learning to the workplace at their own site [39,40].

Overall, participants evaluated their training experience with the MTU as positive. There were no statistically significant differences in evaluations between those who received training remotely versus those who received it face-to-face. Participants felt that the MTU could play an important role in rural medical training, they indicated that they were satisfied with their overall experience in the MTU, and they would recommend the MTU to their colleagues for SBME.

The primary limitation of this study is the relatively small sample size and the inclusion of research subjects from a single institution. However, several things help make the study more robust: (1) the inclusion of a control group; (2) the study design including pretest, posttest, and retention tests; and (3) the triangulation of the results of the modified OSATS checklist and GRS scores with 2 blinded raters demonstrating a favorable interrater reliability provides reassurance of the robustness of the study results [41]. The second limitation is the fact that the physician who was involved in the design of the MTU is the one who led the training sessions for all subjects. It would be interesting to examine the impact on training if a physician not directly involved in the study delivered the training.

There are a number of implications for future SBME and research. First, there is a shift from delivery of medical education in large urban academic centers toward distributed medical education. Technologies such as video conferencing and digital library collections have enabled this advancement and are tied to social, health, and economic benefits [42,43]. There is potential for the MTU concept to play a role in this area, and further research is needed to determine how best to incorporate this concept into practice. Here, it would be particularly important to consider 2 significant time challenges faced by rural practitioners; the maintenance of busy clinical practice, often with limited backup, in addition to the invaluable contributions made in teaching a variety of learners, often with limited resources. A collaborative approach, drawing on local expertise, along with distance-guided mentorship could facilitate valuable advances. The second and related contribution is the potential MTU-enabled cost-savings for trainees and mentors. Cost is often a major barrier to accessing SBME, but it is often not considered in SBME research [44]. Traditional delivery models will have a course, and associated expenses brought to a particular site, such as the related costs of travel, equipment, mentors, and time. The alternative being that the rural practitioner must travel to a central location to teach and is left to address the challenges of patient coverage, time off, and expenses relating to the training and travel. By making cost an important consideration in the development of the MTU, the intention is to make this novel approach more accessible. An economic impact evaluation relating to the use of the MTU in practice is recommended. Third, further studies should be conducted to validate the utility and effectiveness of the MTU concept for skills training that is important to the practice needs of the target audience. Through collaborative discussion and targeted needs assessments with rural practitioners, the specific clinical and educational needs would best be determined. This would enable the examination of Kirkpatrick’s *behavior* (and Miller’s *does*), as well as the *results* levels of the learning evaluation model. As a broader range of skills sessions are delivered remotely through mobile telesimulation, opportunities to study validity and reliability will be more readily available. Fourth, further exploration of skill and scenario characteristics that make them amenable to the remote-mentoring approach to training is necessary, including ability to observe key performance features and maneuvers. This study demonstrated equivalent learning outcomes on assessment of procedural skills for chest tube insertion. This should be further explored for other procedural skills. Fifth, training sessions for this study were conducted in areas with reliable, high-speed internet access; however, rural and remote areas may have limited internet connectivity, which will impede the delivery of remote training and may particularly affect how learners perceive and rate their remote mentoring experience. Future research will explore the use of purpose-built efficient communications systems designed for low bandwidth. Sixth, as proposed by others [3], future research should compare different forms of simulation. Using mobile telesimulation, this would involve comparison of training delivered remotely in an MTU using different levels of fidelity simulators. Finally, the unavailability of mentors comfortable with using simulation-based teaching delivered through telecommunication may present a barrier to expanding this novel approach to SBME [45]. Therefore, the use of the MTU for the remote assessment of skills should also be examined, especially in domains that are poorly covered by traditional written and oral examinations.

### Conclusions

SBME is a well-established training approach, particularly for high-acuity, low-occurrence procedures and scenarios. Practitioners located in rural and remote locations particularly stand to benefit as they face a number of unique challenges with respect to simulation resources, including geographic, cost, and time constraints. This study describes an evaluation of educational efficacy comparing remote versus face-to-face mentoring for procedural skills training. To our knowledge, this study is one of a few to develop and assess SBME combining the concepts of telesimulation and mobile simulation.

We used a conceptual framework based on the combination of Kirkpatrick’s Learning Evaluation Model and Miller’s Clinical Assessment Framework to guide the study. We found that training delivered remotely through the MTU is an effective way to conduct a skills session. Those who were remotely trained had comparable learning outcomes (*shows how*) to subjects who received face-to-face instruction. Participants were also satisfied (*reaction*) with their learning and training experiences. Such remote mentor–led SBME expands opportunities for health practitioners to more easily access the training and mentor-guided practice that they require. Future investigation is needed to examine the utility of the MTU approach in practice, with different skills and level of fidelity, and as a means to provide remote assessment of skills.

## Appendix

Multimedia Appendix 1 Consolidated Standards of Reporting Trials flow diagram.

Multimedia Appendix 2 Differences between pre, post, and retention procedural skills knowledge tests (written).

Multimedia Appendix 3 Modified Objective Structured Assessment of Technical Skills checklist and Global Rating Scale assessment of chest tube performance, mean (standard deviation) reported.

Multimedia Appendix 4 Differences between pre, post, and retention-modified Objective Structured Assessment of Technical Skills checklist and Global Rating Scale test scores.

Multimedia Appendix 5 CONSORT‐EHEALTH checklist (V 1.6.1).

## Abbreviations

GRS: Global Rating Scale
ICC: intraclass correlation coefficient
MTU: mobile telesimulation unit
NLN: National League of Nursing
OSATS: Objective Structured Assessment of Technical Skills
SBME: simulation-based medical education
