# Supplementary figures and images for: Evaluation of a Mobile Telesimulation Unit to Train Rural and Remote Practitioners on High-Acuity Low-Occurrence Procedures: Pilot Randomized Controlled Trial
Source: J Med Internet Res. 2019 Aug 6;21(8):e14587. doi: 10.2196/14587 (PMC6701160; doi:10.2196/14587)

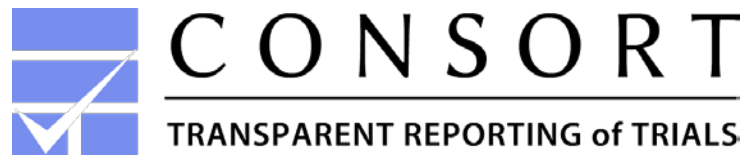

## CONSORT 2010 Flow Diagram

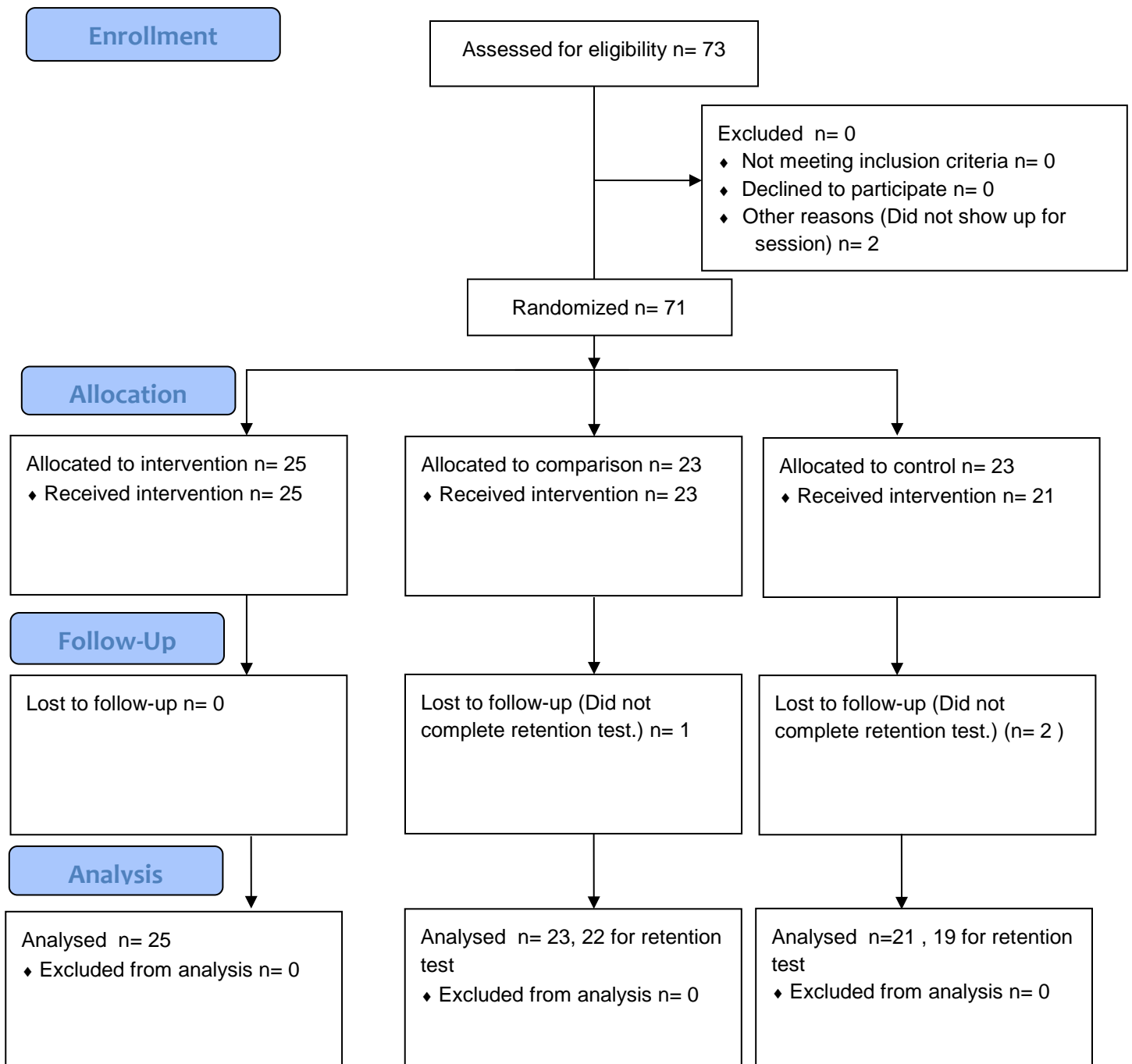

Supplement: Multimedia Appendix 1 [file jmir_v21i8e14587_app1.pdf]
