# Supplementary material for: Evaluation of a Mobile Telesimulation Unit to Train Rural and Remote Practitioners on High-Acuity Low-Occurrence Procedures: Pilot Randomized Controlled Trial
Source: J Med Internet Res. 2019 Aug 6;21(8):e14587. doi: 10.2196/14587 (PMC6701160; doi:10.2196/14587)
Supplement: Multimedia Appendix 2 [file jmir_v21i8e14587_app2.pdf]

Multimedia Appendix

This is a Multimedia Appendix to a full manuscript published in the J Med Internet Res. For full copyright and citation information see http://dx.doi.org/10.2196/jmir.14587

Differences between pre, post and retention procedural skills knowledge tests (written)

	$\chi^2(2)$	*P value*	Mean Rank for Groups		
			**Intervention**	**Comparison**	**Control**
Post minus pre-test score	4.15	.13	-0.50	0.58	0.67
Retention minus post test score	1.55	.46	-2.08	-0.92	-0.83
