# Supplementary material for: Evaluation of a Mobile Telesimulation Unit to Train Rural and Remote Practitioners on High-Acuity Low-Occurrence Procedures: Pilot Randomized Controlled Trial
Source: J Med Internet Res. 2019 Aug 6;21(8):e14587. doi: 10.2196/14587 (PMC6701160; doi:10.2196/14587)
Supplement: Multimedia Appendix 3 [file jmir_v21i8e14587_app3.pdf]

**Multimedia Appendix**

This is a Multimedia Appendix to a full manuscript published in the J Med Internet Res. For full copyright and citation information see http://dx.doi.org/10.2196/jmir.14587

**Modified OSATS Checklist and GRS assessment of chest tube performance, average (SD) reported.**

| | **Intervention group (n=25)** | **Comparison group (n=23)** | **Control group (n= 21)** |
|---|---|---|---|
| Checklist score (maximum= 9) | | | |
| Pre-test | 3.00 (1.80) | 2.96 (1.64) | 2.91 (2.02) |
| Post-test | 6.56 (1.39) | 6.22 (1.24) | 3.62 (2.29) |
| Retention test | 6.16 (1.37) | 5.48 (1.92) | 3.24 (2.05) |
| GRS score (maximum= 35) | | | |
| Pre-test | 10.80 (4.50) | 10.52 (5.51) | 11.14 (4.49) |
| Post-test | 21.48 (6.95) | 21.00 (6.30) | 13.38 (7.21) |
| Retention test | 21.56 (7.43) | 18.39 (8.09) | 12.14 (5.89) |

Checklist was based on 9 questions that were scored as either 0 (incorrect) or 1 (correct), for a possible total of 9 points.

GRS was based on 7 questions with scores on a scale from 1 (worst) to 5 (best), the total was added for a possible total of 35 points.
