# Supplementary material for: Evaluation of a Mobile Telesimulation Unit to Train Rural and Remote Practitioners on High-Acuity Low-Occurrence Procedures: Pilot Randomized Controlled Trial
Source: J Med Internet Res. 2019 Aug 6;21(8):e14587. doi: 10.2196/14587 (PMC6701160; doi:10.2196/14587)
Supplement: Multimedia Appendix 4 [file jmir_v21i8e14587_app4.pdf]

Multimedia Appendix

This is a Multimedia Appendix to a full manuscript published in the J Med Internet Res. For full copyright and citation information see http://dx.doi.org/10.2196/jmir.14587

Differences between pre, post and retention modified OSATS checklist and GRS test scores.

	$\chi^2(2)$	*P value*	Mean Rank for Groups			Pairwise Comparisons
			Inter-vention	Compar-ison	Control	
**Checklist**						
Post minus pre-test score	24.26	<.001	4.00	3.00	1.00	Control-Comparison, P<.001<br>Control-Intervention, P<.001<br>Comparison-Intervention, P=1.00
Retent-ion minus post test score	0.57	.75	-1.00	-1.00	-1.00	n/a
**GRS**						
Post minus pre-test score	16.01	<.001	13.00	12.00	8.00	Control-Comparison,<br>P= .02<br>Control-Intervention,<br>P= .01<br>Comparison-Intervention,<br>P= 1.00
Retent-ion minus post-test score	0.67	.72	0.00	-1.00	-1.00	n/a
